# Total Left Subclavian Artery Occlusion Causing Coronary Subclavian Steal Syndrome and Reversible Left Ventricular Systolic Dysfunction

**DOI:** 10.7759/cureus.110136

**Published:** 2026-06-02

**Authors:** Cody B King, Frank D Russo

**Affiliations:** 1 Internal Medicine, Keesler Medical Center, Biloxi, USA; 2 Cardiology, Keesler Medical Center, Biloxi, USA

**Keywords:** ankle brachial indices, coronary angiography, coronary artery bypass graft, coronary subclavian steal syndrome, heart failure, peripheral artery disease, peripheral interventions

## Abstract

Coronary subclavian steal syndrome (CSSS) is a rare phenomenon that occurs due to severe stenosis of the subclavian artery, resulting in retrograde vertebral and left internal mammary artery (LIMA) flow.

We present a 60-year-old man with severe peripheral artery disease (PAD) and coronary artery bypass graft (CABG) involving the LIMA-left anterior descending artery (LAD) who presented with left arm claudication, vertigo, angina, and heart failure.

This case highlights a patient with a complex set of symptoms across multiple organs with one unifying culprit in the left subclavian artery. Prompt identification and treatment of a total subclavian occlusion resulted in restoration of vertebral and LIMA-LAD flow with resolution of angina, vertigo, and left ventricular systolic dysfunction.

Coronary subclavian steal syndrome is an underrecognized diagnosis that should be on the differential for patients with inter-arm pressure differences and a history of CABG involving the LIMA-LAD. Routine surveillance should be pursued in high-risk patients. Early detection and treatment may improve symptoms and potentially contribute to recovery of left ventricular systolic dysfunction in select patients.

## Introduction

Coronary subclavian steal syndrome (CSSS) is a rare vascular complication that occurs due to stenosis of the proximal subclavian artery, resulting in retrograde flow through the vertebral and left internal mammary artery (LIMA). This phenomenon occurs most commonly in individuals with a known history of coronary artery bypass graft (CABG), specifically utilizing the LIMA-left anterior descending (LIMA-LAD) graft [1]. Risk factors include patients with significant atherosclerotic disease, particularly those with peripheral artery disease (PAD) and cigarette use.

The hemodynamic change that occurs in CSSS is retrograde flow through the vertebral artery and LIMA graft “stealing” blood from the LAD [1]. Blood flows through the path of least resistance [2]. When stenosis occurs at the proximal portion of the subclavian artery, anterograde flow is unable to effectively supply itself or other branches. Hence, the retrograde flow through its branches, such as the vertebral artery and LIMA, occurs [1,2].

Coronary subclavian steal syndrome remains largely underrecognized due to its highly variable presentations. Patients will often present with an interarm blood pressure differential. Symptoms include vertebrobasilar insufficiency, such as dizziness, imbalance, syncope, and ataxia. Furthermore, the retrograde flow via the LIMA-LAD graft causes cardiovascular symptoms like angina, myocardial ischemia, and possibly left ventricular systolic dysfunction [3,4]. Prolonged myocardial ischemia from CSSS may lead to myocardial stunning and, conceivably, left ventricular systolic dysfunction.

Currently, there are no formal guidelines for monitoring patients post-CABG [5]. CSSS can be closely surveilled with minimally invasive interarm blood pressure measurements, which can be suggestive of subclavian stenosis [1]. In high-risk patients’ post-CABG, interarm blood pressure differentials can be helpful to physicians as a surveillance tool for early detection of CSSS.

We present a rare case of CSSS with a newly reduced ejection fraction (EF) that was successfully treated with percutaneous intervention (PCI). This case underscores the need for routine monitoring of high-risk patients’ status post CABG. Early recognition and treatment may prevent symptom progression, recover a reduced ejection fraction, and improve quality of life.

This case was previously presented as a poster at the Annual American College of Cardiology meeting in New Orleans in March 2026.

## Case presentation

History of presentation

**Figure 1:**
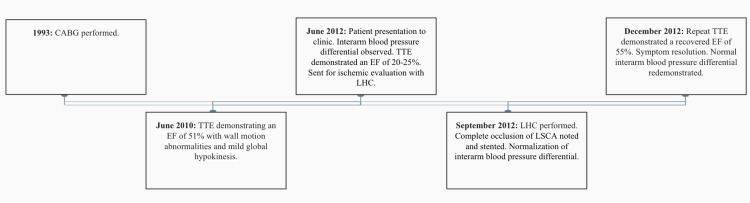
Timeline of the patient course from initial CABG, presentation, and eventual intervention with resolution of symptoms and recovered ejection fraction. CABG: coronary artery bypass graft; TTE: transthoracic echocardiogram; LHC: left heart catheterization; LSCA: left subclavian artery; EF: ejection fraction

A 60-year-old man who presented to the clinic with left arm claudication, vertebrobasilar symptoms, angina, and heart failure (ejection fraction (EF) 20-25%). Patient symptoms progressed over several months to years (Figure 1). The patient was experiencing intermittent dizziness and presyncope/syncopal episodes. There were no other concerning features such as dysarthria, diplopia, or ataxia. Furthermore, he had noticed periods of angina unrelieved by anti-anginal therapy and left arm exertional claudication that would last for multiple minutes before resolution. He also had dyspnea on exertion and lower extremity edema, which had limited his ability to accomplish activities of daily living and frequently required him to rest.

On physical exam, the patient had evidence of sparse hair growth on the left arm and was cooler to the touch than the right arm. The left arm also had a diminished pulse and a delayed capillary refill. There was a significant interarm pressure differential of 40 mmHg systolic between the right (112/76) and left upper extremities (72/58).

Past medical history

The patient’s past medical history includes hypertension (HTN), hyperlipidemia (HLD), severe peripheral artery disease (PAD) and coronary artery disease (CAD) status post coronary artery bypass graft (CABG).

The patient has a history of severe PAD with persistent lower extremity claudication symptoms despite previous intervention with aortobifemoral bypass. His symptoms have progressed rapidly to include not only the lower extremities but also the left upper extremity. Despite numerous attempts at counseling for tobacco cessation, this patient has continued cigarette smoking.

The patient’s baseline medications included aspirin, rosuvastatin, metoprolol succinate, and lisinopril. Unfortunately, he did have issues with medication adherence, given multiple medications with refills and expired prescriptions noted during his medication reconciliation.

He had a two-vessel CABG performed in 1993, including the LIMA to the LAD and saphenous vein graft to the right coronary artery (RCA). The patient has no known history of heart failure. Previous transthoracic echocardiograms (TTE) demonstrated a preserved ejection fraction of 51% (Figure 1).

Differential diagnosis

The differential diagnosis included: progression of PAD, worsening CAD of the native vessels, aortic dissection, valvular disease, large vessel vasculitis, thoracic outlet syndrome, and coronary subclavian steal syndrome (CSSS).

Aortic dissection was ruled out given there was no evidence of hemodynamic compromise or dissection on transthoracic echocardiogram (TTE). Valvular disease was excluded by TTE and would be less likely to cause interarm blood pressure differentials by itself. Furthermore, any large vessel vasculitis was considered less likely given this patient’s age and absence of other signs of systemic inflammation.

Thoracic outlet syndrome was ruled out given the lack of dermatomal distribution to the left arm. He did not have any vessels/nerves being compressed on imaging by a clavicular bone or rib.

Worsening of CAD, PAD, and CSSS was evaluated further by angiography. Angiography allowed visualization of flow abnormalities that would help to differentiate between these processes. Worsening of CAD and PAD both would not show a retrograde flow pattern from the vertebral and coronary artery via the LIMA-LAD graft as seen with CSSS.

Investigations and management

A TTE was performed to assess if a new cardiomyopathy or valvular disease could explain his symptoms. The TTE demonstrated a severely reduced EF of 20-25% with anterior hypokinesis via the modified Simpson’s biplane method. No contrast was used during the study. No arrhythmia or tachycardia was observed. The results of the echocardiogram, interarm blood pressure differential, and the patient’s symptoms were highly suspicious for progression of coronary disease and left upper extremity stenosis. A left heart catheterization and angiography were scheduled.

**Figure 2:**
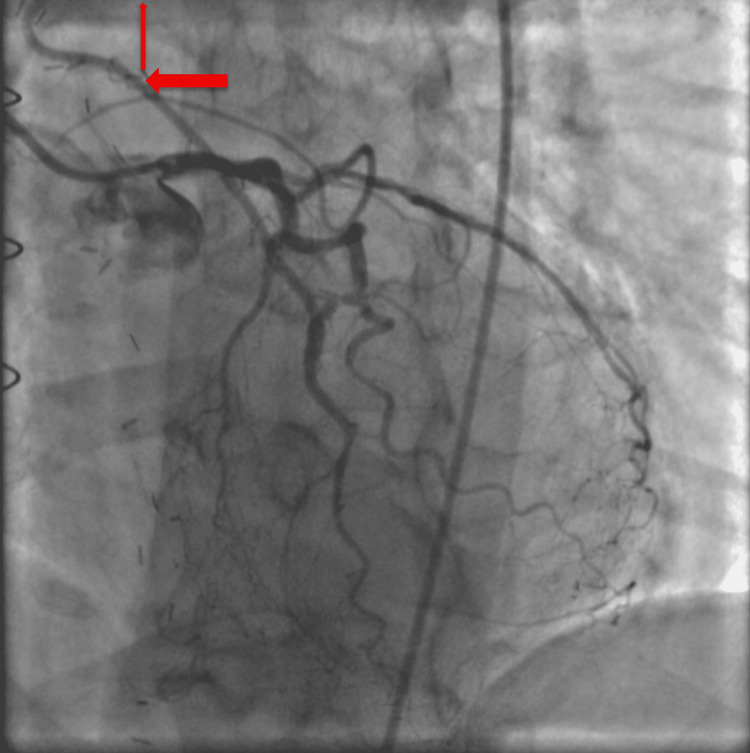
Retrograde blood flow through the LIMA demonstrated on angiography. This figure shows the anatomy of the LIMA graft (location noted by the wide red arrow) connecting to the LAD. The directionality of flow during angiography is demonstrated by the skinny red arrow. LIMA:  left internal mammary artery

The right femoral artery was accessed. A JL4 catheter was used to cannulate the left main coronary artery. There was significant stenosis of the LAD in the proximal to mid portion, measuring roughly 90%. The LIMA was opacified with retrograde flow with left angiography (Figure 2).

**Figure 3:**
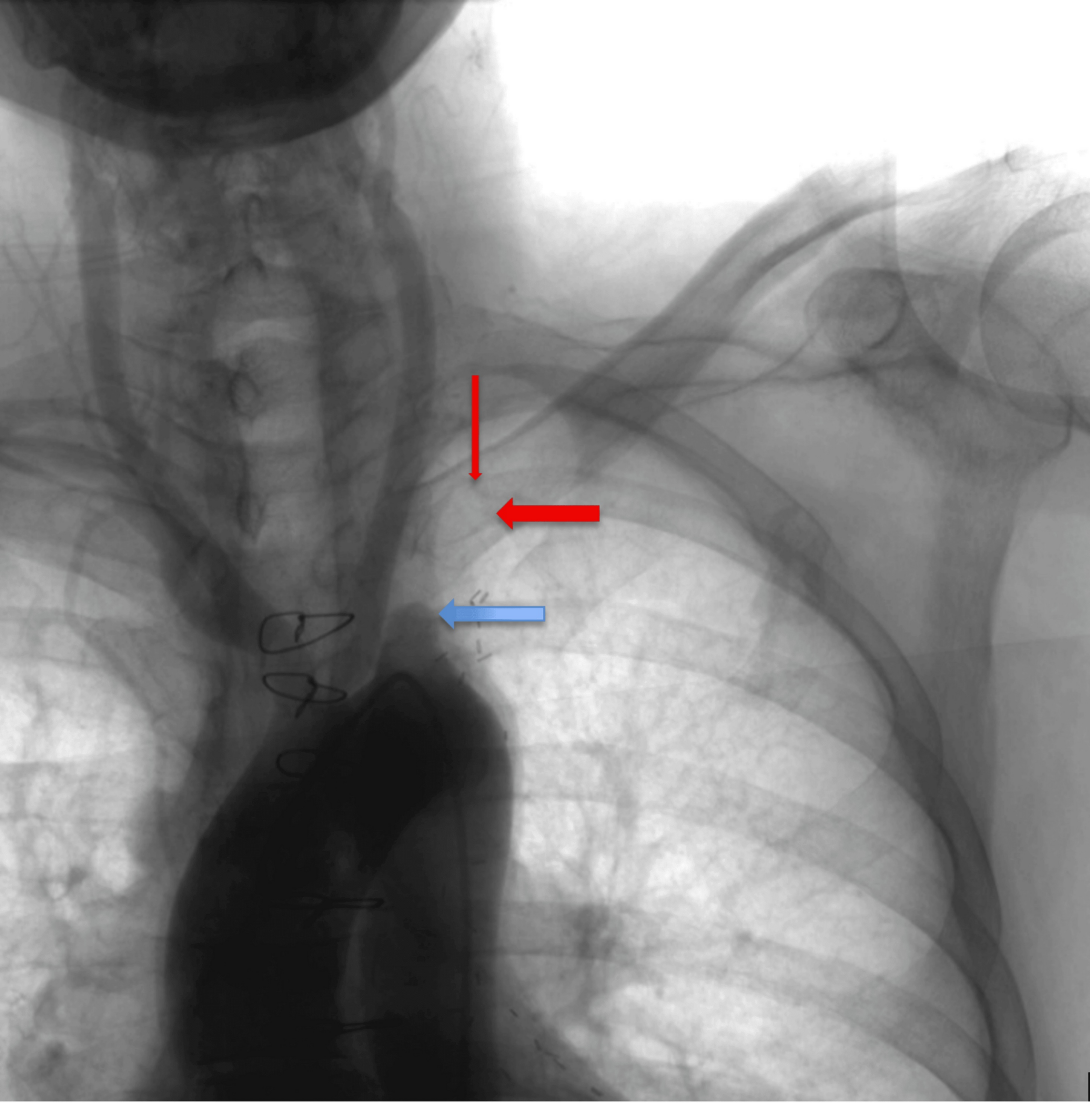
Angiography showing complete occlusion of the proximal left subclavian artery (noted by the blue arrow). This figure shows the rough anatomy of the LIMA (estimated location noted by the wide red arrow) and the retrograde direction of flow noted by the skinny red arrow. LIMA:  left internal mammary artery

**Figure 4:**
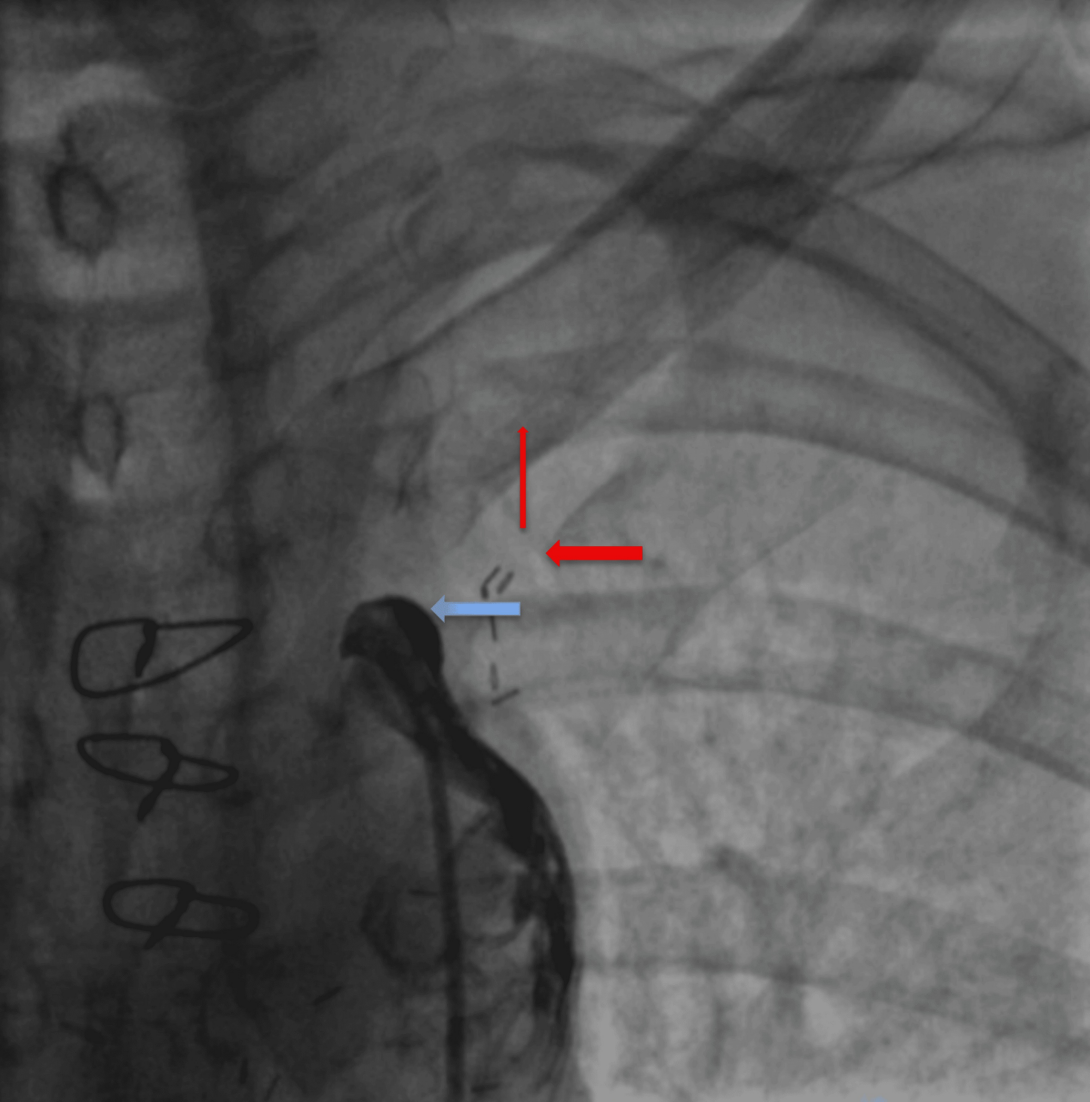
Angiography showing complete occlusion of the proximal left subclavian artery (noted by the blue arrow). This figure shows the rough anatomy of the LIMA (estimated location noted by the wide red arrow) and the retrograde direction of flow noted by the skinny red arrow. LIMA:  left internal mammary artery

A JR4 was used to assess the right coronary artery and its associated grafts, which were largely unrevealing. The JR4 was then used to select the left subclavian artery (LSCA) to further visualize the LIMA, which demonstrated 100% occlusion of the subclavian artery at its proximal position (Figures 3, 4). There was retrograde flow through the vertebral artery and LIMA-LAD (Figures 2, 4), evidence for combined coronary and subclavian steal syndrome.

**Figure 5:**
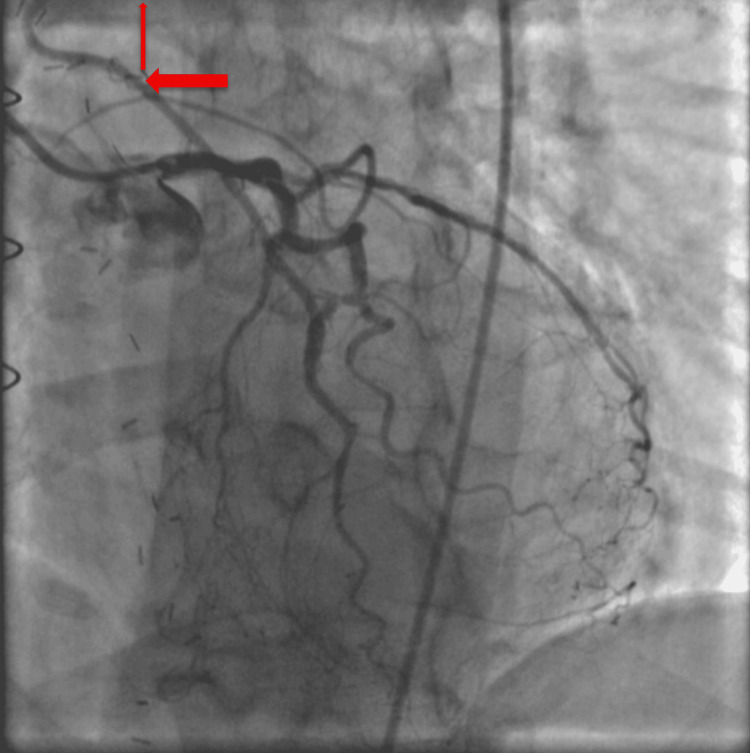
Retrograde blood flow through the LIMA demonstrated on angiography. This figure shows the anatomy of the LIMA graft (location noted by the wide red arrow) connecting to the LAD. The directionality of flow during angiography is demonstrated by the skinny red arrow. LIMA:  left internal mammary artery; LAD: left anterior descending artery

**Figure 6:**
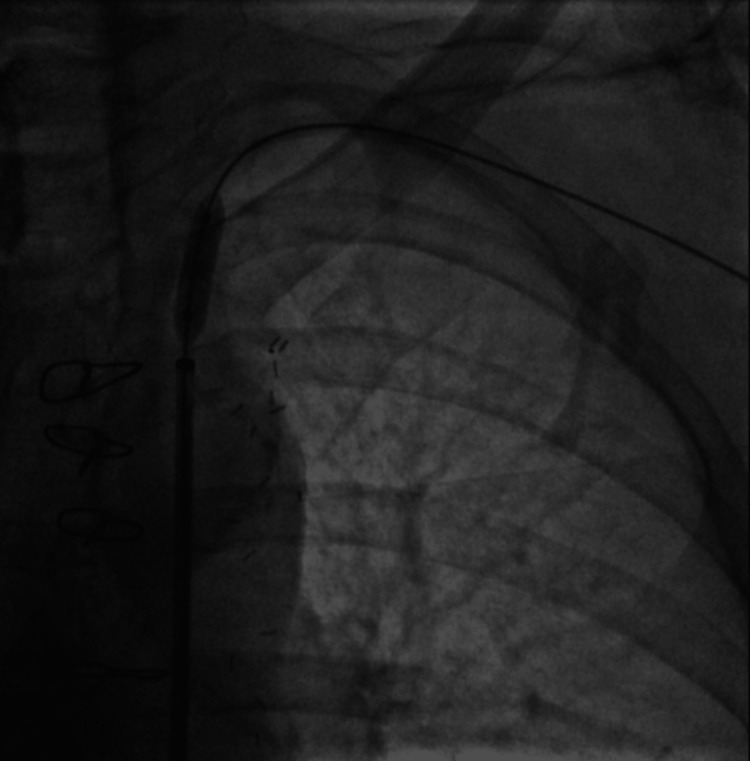
Endovascular intervention with angioplasty and balloon expandable stent placement to the left subclavian artery.

**Figure 7:**
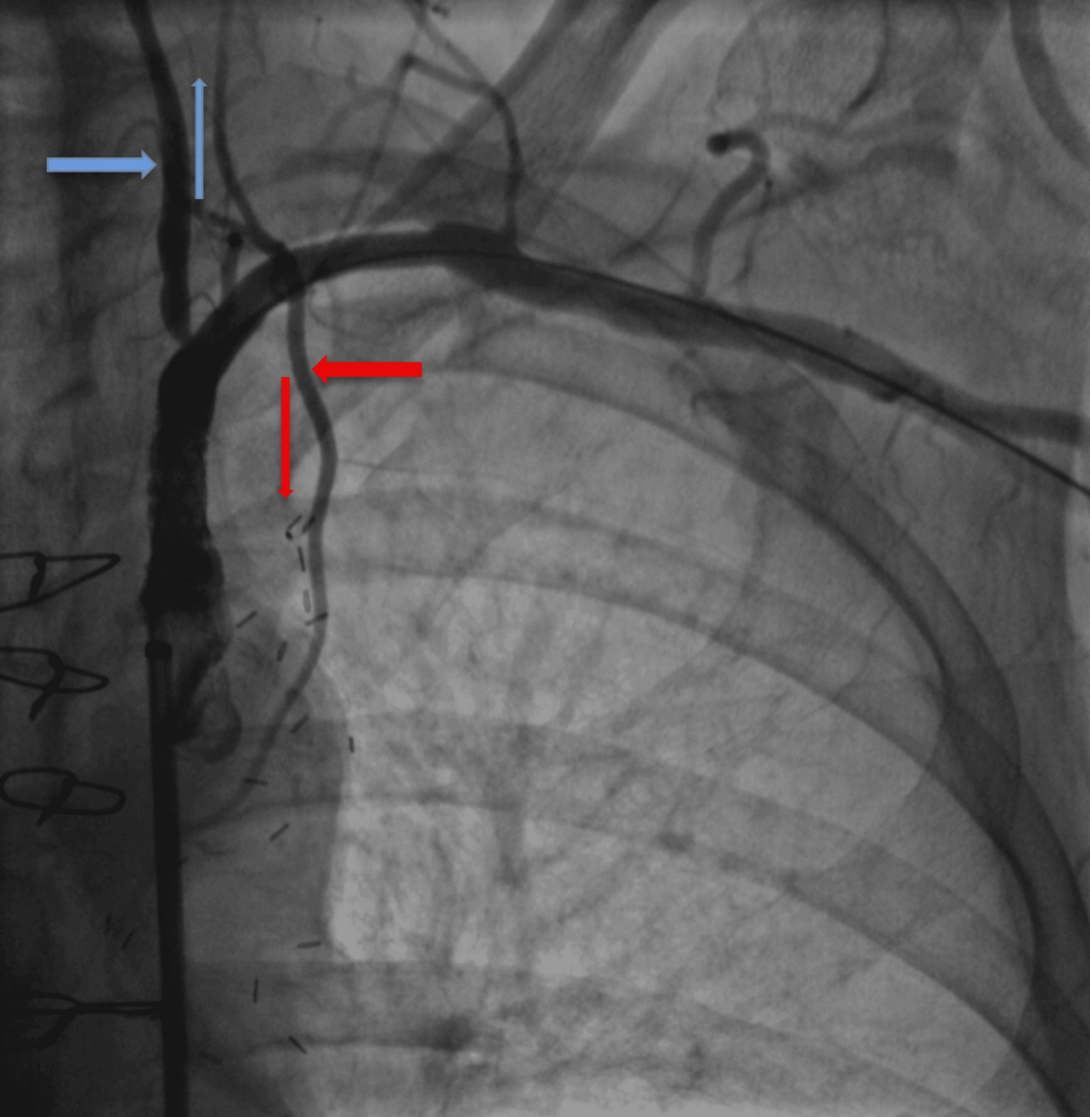
Endovascular intervention with angioplasty and balloon expandable stent placement to the left subclavian artery with full revascularization. This figure demonstrates revascularization of the left proximal subclavian artery with restoration of anterograde flow through the vertebral artery and LIMA. The vertebral artery is indicated by the wide blue arrow, and the new directionality of flow (anterograde) after stent placement is shown by the skinny blue arrow. The LIMA is indicated by the wide red arrow. Anterograde flow through the LIMA after stenting is demonstrated by the skinny red arrow. LIMA:  left internal mammary artery

Upon discovery of the occlusion during coronary angiography, heparin was loaded, a stiff-angled guidewire, and a quick-cross catheter were used to cross the lesion (Figure 5). The 100% occlusion was dilated with a 6.0 x 20 mm REEF balloon at 12 atmospheres, and a 7.0 x 30 mm Assurant balloon-expandable stent was placed at 10 atmospheres (Figure 6). Follow-up angiography revealed an excellent result with 0% residual stenosis and anterograde blood flow (Figure 7). There were no post-procedural complications.

Outcome and follow-up

The patient had a close follow-up two weeks after successful stent placement. He has been adherent to his dual antiplatelet therapy and has had no recurrence of his claudication and vertebrobasilar symptoms. There was improvement in the interarm blood pressures post-procedurally and at follow-up (Figure 1). Left upper extremity blood pressure was measured at 116/76, and the right upper extremity was 117/70. The patient had no other changes to his medical therapy. Follow-up TTE using the modified Simpson’s method three months after the procedure showed improvement of the patient’s ejection fraction from 20-25% to 55%.

## Discussion

This case describes a patient with multiple symptoms that can be attributed to a single unifying diagnosis, CSSS. CSSS should be suspected in patients with a history of CABG presenting with left arm claudication, vertebrobasilar insufficiency, and a significant upper extremity blood pressure differential. Understanding the pathophysiology and risk factors associated with this disease process is crucial for earlier recognition and intervention. Prompt revascularization can have a major impact on the patient’s quality of life.

Coronary subclavian steal syndrome occurs when significant atherosclerotic plaque build-up causes proximal occlusion of the subclavian artery. This phenomenon can occur in individuals with a known history of CABG involving the LIMA as well as other risk factors, including known PAD, tobacco use disorder, and diabetes [1]. When significant stenosis occurs at the proximal subclavian artery, retrograde blood flow or “steal” occurs via the vertebral artery and LIMA [3].

The challenge that many physicians face with CSSS is early recognition, often due to low clinical suspicion as a result of presentation variability. CSSS can present on a spectrum from being asymptomatic to causing myocardial ischemia or cardiogenic shock [3,4]. Nevertheless, if there is clinical suspicion for CSSS, testing should be pursued. In particular, interarm blood pressure differentials can be a helpful tool towards diagnosis, as studies have shown that evidence of PAD and a bilateral upper extremity blood pressure differential of ≥10-15 mmHg can be specific for subclavian stenosis [1]. Other alternatives that can aid in diagnosis are computed tomography (CT), chest angiography, doppler/duplex ultrasound, or magnetic resonance angiography (MRA) [5]. According to the American College of Cardiology/American Heart Association (AHA/ACC) guidelines, the gold standard of diagnosis and treatment of subclavian stenosis, such as CSSS, is an endovascular approach [6].

The majority of patients with clinically significant CSSS require intervention, whether surgically with carotid-subclavian bypass or via revascularization with stenting. Research comparing these treatment modalities is limited; however, most studies recommend percutaneous revascularization with stenting given high success, low complication risk, and high patency rates [7-9]. A balloon-expandable stent is preferred because it allows precise positioning [8,9]. Ultimately, percutaneous intervention is the preferred modality of choice, as CSSS is commonly identified late in its disease course, causing significant symptoms and end-organ dysfunction.

The main organ systems affected by CSSS include the neurological and cardiovascular systems. It causes vertebrobasilar insufficiency symptoms as well as angina, myocardial ischemia causing left ventricular systolic dysfunction, and upper extremity claudication, as seen in our case [10-12]. The recovered ejection fraction after stenting has been documented in limited cases. However, given the reversal of hemodynamics caused by CSSS, it is hypothesized that myocardial stunning from continued myocardial ischemia caused the development of left ventricular systolic dysfunction [10-12]. A similar mechanism is likely at play in the case presented. It is important to consider, however, that there may have been a degree of over/under estimation due to reader and technician variability in analyzing both the initial and post-intervention TTE. Furthermore, the authors cannot entirely exclude other potential confounding factors towards the reduction in EF alongside the CSSS.

There are currently no formal guidelines for surveillance of CSSS post-CABG. At the authors’ institution, patient-centered surveillance is implemented for high-risk patients. This includes both pre- and post- intervention interarm blood pressure measurements. Patients with known risk factors, baseline bilateral upper extremity blood pressures can be established and measured routinely following the procedure. Interarm blood pressure differentials can be tracked in addition to symptoms to further stratify patients for further testing and if needed, revascularization.

## Conclusions

Coronary-subclavian steal syndrome is a rare disease process that often goes underdiagnosed and untreated. The clinical significance of subclavian stenosis should not be overlooked as it causes a multitude of symptoms across multiple organ systems. In the case presented, a single culprit lesion was discovered decades after CABG and successfully treated with percutaneous intervention, resulting in complete resolution of symptoms, normalization of interarm blood pressure differential, and recovery of a low ejection fraction. The authors acknowledge that restoration of LIMA-LAD flow did demonstrate recovery of a low ejection fraction and symptoms; however, other confounding contributors towards this finding cannot be entirely excluded.

There are currently no guidelines for monitoring patients’ status post CABG. However, routine surveillance can be considered with baseline interarm blood pressure measurements. Early recognition and treatment may improve symptoms and could possibly lead to recovery of left ventricular systolic function in patients with CSSS.

## References

[1] English JA, Carell ES, Guidera SA, Tripp HF (2001). Angiographic prevalence and clinical predictors of left subclavian stenosis in patients undergoing diagnostic cardiac catheterization. Catheter Cardiovasc Interv.

[2] Cua B, Mamdani N, Halpin D, Jhamnani S, Jayasuriya S, Mena-Hurtado C (2017). Review of coronary subclavian steal syndrome. J Cardiol.

[3] Monteagudo-Vela M, Bastante T, Monguió-Santín E, Del Val D, Panoulas V, Reyes-Copa G (2023). Coronary-subclavian steal syndrome: a case report of a rare entity that can become a deadly threat. Eur Heart J Case Rep.

[4] Real C, Vivas D, Martínez I (2021). Endovascular treatment of coronary subclavian steal syndrome: a case series highlighting the diagnostic usefulness of a multimodality imaging approach. Eur Heart J Case Rep.

[5] Gerhard-Herman MD, Gornik HL, Barrett C (2017). 2016 AHA/ACC guideline on the management of patients with lower extremity peripheral artery disease: executive summary: a report of the American College of Cardiology/American Heart Association Task Force on Clinical Practice Guidelines. J Am Coll Cardiol.

[6] Hillis LD, Smith PK, Anderson JL (2011). 2011 ACCF/AHA Guideline for Coronary Artery Bypass Graft Surgery: executive summary: a report of the American College of Cardiology Foundation/American Heart Association Task Force on Practice Guidelines. Circulation.

[7] Jahic E, Avdagic H, Iveljic I, Krdzalic A (2019). Percutaneous transluminal angioplasty of subclavian artery lesions. Med Arch.

[8] Gareeb J, Ahmed FJ, Mohammad HH (2025). Early onset of coronary subclavian steal syndrome: a case report and literature review. Barw Med J.

[9] Satti SR, Golwala SN, Vance AZ, Tuerff SN (2016). Subclavian steal: endovascular treatment of total occlusions of the subclavian artery using a retrograde transradial subintimal approach. Interv Neuroradiol.

[10] Ambesh P, Sawalha K, Groudan K, Lotfi A, Giugliano G (2021). Coronary subclavian steal syndrome causing myocardial Infarction. Ann Card Anaesth.

[11] Takach TJ, Reul GJ, Cooley DA, Duncan JM, Livesay JJ, Ott DA, Gregoric ID (2006). Myocardial thievery: the coronary-subclavian steal syndrome. Ann Thorac Surg.

[12] Bryan FC, Allen RC, Lumsden AB (1995). Coronary-subclavian steal syndrome: report of five cases. Ann Vasc Surg.

